# readat: An R package for reading and working with SomaLogic ADAT files

**DOI:** 10.1186/s12859-016-1007-8

**Published:** 2016-05-04

**Authors:** Richard J. Cotton, Johannes Graumann

**Affiliations:** Proteomics Core, Weill Cornell Medicine - Qatar, PO Box 24144, Doha, State of Qatar

**Keywords:** SomaLogic, Proteomics, Dynamic range, ADAT, R, Bioconductor, Software

## Abstract

**Background:**

SomaLogic’s SOMAscan™ assay platform allows the analysis of the relative abundance of over 1300 proteins directly from biological matrices such as blood plasma and serum. The data resulting from the assay is provided in a proprietary text-based format not easily imported into R.

**Results:**

*readat* is an R package for working with the SomaLogic ADAT file format. It provides functionality for importing, transforming and annotating data from these files. The package is free, open source, and available on Bioconductor and Bitbucket.

**Conclusions:**

*readat* integrates into both Bioconductor and traditional R workflows, rendering it easy to make use of ADAT files.

**Electronic supplementary material:**

The online version of this article (doi:10.1186/s12859-016-1007-8) contains supplementary material, which is available to authorized users.

## Background

SOMAscan^TM^ [1] is an aptamer-based array from SomaLogic (Boulder, Colorado) for affinity-proteomic analysis which allows simultaneous measurement and quantitation of over 1300 proteins directly from biological matrices such as blood. Proteins targeted include very low abundance proteins as cytokines, chemokines, and interleukins which, due to dynamic range limitations, are particularly challenging to access using mass spectrometry-based proteomics.

Experimental data resulting from the assay is provided by SomaLogic in a proprietary text-based format called ADAT. The company provides a software suite for working with these files, but no free, open source solution currently exists to access the data contained in them.

## Implementation

*readat* is an R [2] package with a GPL-3 licence, and is designed to easily integrate into existing R/Bioconductor workflows. The package provides functionality for importing data from ADAT files, transforming it in various useful ways, and retrieving additional annotation.

### The ADAT file format

ADAT is a tab-delimited text file format. The contents include SOMAmer^Ⓡ^ (Slow Off-rate Modified Aptamer) reagent intensities, sample data, sequence data, experimental metadata, and a checksum. Since all these data types appear in the same file, the use of standard functions for reading tab-delimited files to import data from this file format is rendered non-trivial.

The file format begins with a first line containing a SHA-1 checksum, allowing the integrity of the file to be verified. This is followed by a line marked ^HEADER, and two columns of key-value experimental metadata. Sections marked ^COL_DATA and ^ROW_DATA specify the fields used for sequence and sample data respectively. Sequence data fields can include SomaLogic’s internal IDs for the SOMAmer reagent and target proteins, protein names, UniProt IDs, Entrez Gene IDs and symbols, and whether or not the sequence’s results passed the quality control tests. Sample data fields can include IDs for the sample, subject, slide and plate, notes on the sample quality, and whether or not the sample’s results passed quality control tests imposed by the supplier. A section marked ^TABLE_BEGIN contains the sequence, sample and intensity data.

### Obtaining *readat*

The stable version of *readat* is available on Bioconductor and can be installed with: 

The development version is available on Bitbucket and can be installed with: 

The source package as it stands at the time of publication is also available online as Additional file 1.

### Data import

The readAdat function imports data from ADAT files. The resultant data variable is an object of class WideSomaLogicData, which consists of a *data.table*, from the package of the same name [3], for the sample and intensity data, and three attributes for the sequence data, metadata, and checksum.

The sequence data, metadata, and checksum values can be retrieved with accessor (“get”) functions, and changed with mutator (“set”) functions.

### Data transformation

The default format is not appropriate for all data analytical needs. When using *ggplot2* [4] or *dplyr* [5], for example, it is more convenient to have one intensity per row rather than one sample per row. The package contains a melt method to transform WideSomaLogicData into LongSomaLogicData.

To further ease integration of ADAT encoded data into existing data analytical workflows, the package also includes a method to convert WideSomaLogicData objects into *Biobase* [6] ExpressionSets.

### Annotation

ADAT files typically contain target protein names, UniProt IDs, Entrez Gene IDs and Entrez Gene symbols for each SOMAmer reagent sequence. Additional IDs and annotation are available via accessor functions to datasets stored in the package. Currently Ensembl IDs, UniProt keywords, chromosomal positions, PFAM IDs and descriptions, KEGG definitions, modules, and pathways, and GO annotations are supported.

## Results

*readat* contains sample datasets probed with both SomaLogic’s 1129 (1.1k) and 1310 (1.3k) suites of SOMAmer reagents. To demonstrate the features of the package, we exhibit the “1.3k” dataset.

The dataset contained in the package represents plasma samples from 20 US adults aged between 35 and 75 years old. It is a subset of a 168 samples cross-sectional cohort of the US population (evenly represented by decile from 35 to 75) collected by Covance (Princeton, NJ), a contract research organisation, under contract to SomaLogic. All analyzed and included data are deidentified and therefore do not require IRB approval. The 20 samples included are split into age groups (“old”, 50 or older; “young”, under 50) and provided by SomaLogic for use in analysis examples and tutorials.

### Import

To import the data, type:



Intensity readings for eleven of the SOMAmer reagents did not pass SomaLogic’s quality control checks, and are excluded on import by default.



The dataset contains ten samples from “young” patients (age 35 to 50) and ten samples from “old” patients (age 50 to 75), split evenly by gender. 

### Reshaping and plotting

To see which sequences display the most difference between, for example, genders it is easier to work with the data in “long” form, with one intensity value per row. This conversion requires access to the melt generic function in the *reshape2* package [7].



*readat* has a convenience function for finding the top sequences with the largest variation between groups. By default it looks for difference in the “SampleGroup” column, which in this case contains genders.



One last piece of data housekeeping is to provide more human-readable names for the sequences.



Now the *ggplot2* package can be used to visualize the differences in intensities between the groups. For larger datasets, boxplots may be more appropriate than the scatterplots shown here.



**Fig. 1:**
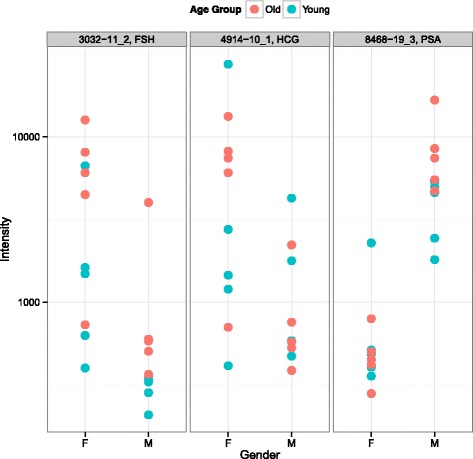
Top differentiators by gender. The three proteins that display the largest difference in intensity between genders are *follicle-stimulating hormone* (FSH), *human chorionic gonadotropin* (HCG), and *prostate-specific antigen* (PSA)

In Fig. 1, *Follicle stimulating hormone* (FSH) and *human chorionic gonadotropin* (HCG) both appear to be more abundant in females, and in particular older females, which is consistent with their function in the ovulatory process [8, 9] and the effects of menopause [10, 11]. *Prostate-specific antigen* (PSA) is more abundant in males, especially older males, as expected by its secretion from prostatic epithelial cells and association with prostate cancer [12].

### ExpressionSets and modelling

For Bioconductor workflows, it is often easier to work with an ExpressionSet object.



To explore differences between genders and age groups, we can define a single variable from the interaction of the individual variables.



The following example uses linear models from the *limma* package [13]. Further explanation can be found in Chapter 8 of the Limma User’s Guide, obtained by running limma::limmaUsersGuide(). *limma* requires the definition of a model design and contrasts.



We can now calculate differential expression via empirical Bayes moderation of the standard errors from linear model fits.



The top differential expression for each contrast, along with its coefficient, is shown below.



For both the “old” and “young” groups, *prostate-specific antigen* (PSA) is the strongest differentiator of genders and mirrors the more simple analysis above. For both genders *growth/differentiation factor 15* (MIC-1) is the strongest age group differentiator. Its age-dependent increase in abundance is consistent with the literature [14].

### Annotation

Additional annotation, for example PFAM IDs, can be retrieved for each SOMAmer reagent using auxilliary functions such as getPfam. By default the function returns a list of data frames; the simplify argument returns the results more concisely as a single data frame.



In the previous example, notice that PFAM IDs are mapped to SOMAmer reagents via Entrez Gene IDs, and several Entrez Gene IDs may be associated with a given SeqId.

### Future developments

The package will continue to track the ADAT file specification as it evolves.

## Conclusions

Affinity proteomic approaches offer dynamic range characteristics and parallelization potential exceeding those of mass spectrometry-based techniques and are thus attractive for the analysis of clinical samples where massive in-sample concentration differences and large cohort size requirements due to human genetic diversity conincide. Among such approaches the nucleic acid based SOMAscan assay by SomaLogic is prominent, as the affinity reagents used are raised with comparative ease by SELEX [15–17] and entirely synthetic, contrasting them to antibodies and other proteinaceous binders, which must be raised and produced in vivo.

*readat* is a free, open source, and easy to use R package that lets you import and work with SomaLogic’s ADAT file format.

## Availability and requirements

- **Project name:** readat
- **Project home page:**https://bitbucket.org/graumannlabtools/readat
- **Operating system(s):** All platforms where R is available, including Windows, Linux, OS X, BSD, Solaris
- **Programming language:** R
- **Other requirements:** R 3.1.2 or higher, and the R packages assertive, Biobase, data.table, dplyr, stringi, and tidyr
- **License:** GNU GPL
- **Any restrictions to use by non-academics:** Freely available to everyone

## Additional file

Additional file 1 R source package of readat at the time of publication.

## Abbreviations

FSH: follicle stimulating hormone
HCG: human chorionic gonadotropin
MIC-1: growth/differentiation factor 15
PFAM: protein FAMilies database
PSA: prostate-specific antigen
SOMAmer: slow off-rate modified aptamer
